# Evaluation of the therapeutic potential of Epigallocatechin-3-gallate (EGCG) via oral gavage in young adult Down syndrome mice

**DOI:** 10.1038/s41598-020-67133-z

**Published:** 2020-06-26

**Authors:** Charles R. Goodlett, Megan Stringer, Jonathan LaCombe, Roshni Patel, Joseph M. Wallace, Randall J. Roper

**Affiliations:** 1 IUPUI Department of Psychology, 402 North Blackford Street, LD 124, Indianapolis, IN 46202-3275 USA; 2 IUPUI Department of Biology, 723 West Michigan Street; SL 306, Indianapolis, IN 46202-3275 USA; 3 IUPUI Department of Biomedical Engineering, 723 West Michigan Street; SL 220B, Indianapolis, IN 46202-3275 USA

**Keywords:** Gene expression, Genotype, Target validation, Learning and memory

## Abstract

Epigallocatechin-3-gallate (EGCG) is a candidate therapeutic for Down syndrome (DS) phenotypes based on *in vitro* inhibition of DYRK1A, a triplicated gene product of Trisomy 21 (Ts21). Consumption of green tea extracts containing EGCG improved some cognitive and behavioral outcomes in DS mouse models and in humans with Ts21. In contrast, treatment with pure EGCG in DS mouse models did not improve neurobehavioral phenotypes. This study tested the hypothesis that 200 mg/kg/day of pure EGCG, given via oral gavage, would improve neurobehavioral and skeletal phenotypes in the Ts65Dn DS mouse model. Serum EGCG levels post-gavage were significantly higher in trisomic mice than in euploid mice. Daily EGCG gavage treatments over three weeks resulted in growth deficits in both euploid and trisomic mice. Compared to vehicle treatment, EGCG did not significantly improve behavioral performance of Ts65Dn mice in the multivariate concentric square field, balance beam, or Morris water maze tasks, but reduced swimming speed. Furthermore, EGCG resulted in reduced cortical bone structure and strength in Ts65Dn mice. These outcomes failed to support the therapeutic potential of EGCG, and the deleterious effects on growth and skeletal phenotypes underscore the need for caution in high-dose EGCG supplements as an intervention in DS.

## Introduction

Epigallocatechin-3-gallate (EGCG), the main polyphenol found in green tea, has been hypothesized to reduce the risk or pathogenesis of several neurodegenerative and cardiovascular diseases, as well as cancer^1–4^. The effects of EGCG or EGCG-containing green tea extracts have been assessed in over 100 clinical trials in a wide-range of diseases and disorders that represent diverse underlying etiologies (search as of December 2019 on Clinicaltrials.gov), consistent with multiple cellular and molecular mechanisms that are thought to be modified by EGCG^5–7^.

EGCG was initially identified as a candidate therapeutic treatment for Down syndrome (DS) phenotypes based largely on the *in vitro* demonstration that EGCG inhibits Dual-specificity tyrosine-phosphorylation regulated kinase 1A (DYRK1A) activity^8,9^. *DYRK1A* is a serine-threonine kinase located on human chromosome 21 (Hsa21) and found in three copies in individuals with Trisomy 21 (Ts21) and in most DS mouse models^10–12^. *DYRK1A* is a dosage-sensitive gene that is particularly active during embryonic and perinatal development^13,14^. Differential expression of *Dyrk1a* in genetically modified mice (both overexpression and underexpression) has been correlated with deleterious phenotypes including cognitive deficits, neurodevelopmental abnormalities, low body weight, and reduced brain size^15,16^. The hypothesis that trisomic *DYRK1A* is a major contributor to DS phenotypes^17,18^ and the demonstration that EGCG inhibits DYRK1A activity *in vitro* has fueled enthusiasm for the hypothesis that EGCG and green tea extracts could be an effective nutraceutical therapy for DS^19^.

Preclinical evaluation of effects of consuming EGCG-containing green tea extracts on DS phenotypes have been assessed in DS mouse models, including Ts65Dn mice that are trisomic for approximately half of the genes found in three copies in Ts21 and mice that are transgenic for *Dyrk1a* (Table 1). Mice have typically been given green tea extracts in drinking water or chow and various improvements have been shown in anatomical, cellular and behavioral traits. Reports of beneficial effects of EGCG-containing green tea extracts in mouse models led to two clinical trials of the therapeutic potential of green tea extracts in individuals with DS. A three-month treatment of green tea extract (45% EGCG, 9 mg/kg/day EGCG) improved performance on a subset of measures on a battery of cognitive tests, including visual recognition memory^20^. A subsequent randomized, placebo-control, double-blind study administered the same supplement (or placebo) to individuals with DS in combination with cognitive training for 12 months with follow-up testing 6 months after treatment ended. The group receiving EGCG displayed significantly higher scores in two of the 15 measures of the battery of cognitive tests (visual recognition immediate memory; inhibitory control) and in one of the nine measures of adaptive behavior^21^. The modest gains in a limited set of measures of cognitive and adaptive function in these clinical trials suggest some benefit of the green tea supplement containing EGCG.

**Table 1:** Preclinical treatment of Down syndrome mouse models with EGCG-containing green tea extracts and/or other formulations.

Mouse model	Sex	Age	EGCG	Treatment duration	Observed Effects	Ref
YACtg152F7	N/A	0–3 months	Green tea infusion (0.6–1 mg/day EGCG) Polyphenon 60 (27% EGCG, 1.2 mg/day EGCG)	Gestation-adulthood	• Normaled brain size • Improved novel object recognition memory	^58^
TgDyrk1A	female	1 month	Mega Green Tea Extract (45% EGCG, 2–3 mg/day EGCG, ~40–60 mg/kg/day EGCG^23^)	1 month	• Reduced DYRK1A kinase activity in the hippocampus • Reduction to WT levels of the density of proliferating (Ki67+) dentate gyrus neural progenitor cells • Normalized proportion of proliferating cells exiting the cell cycle	^59^
mBACtgDyrk1a	male	4–6 months	LifeExtension For Longer Life (45% EGCG, 120–200 mg/kg/day EGCG)	4–6 weeks	• Restored spine density • Improved long term potentiation • Normalized associated biochemical markers	^54^
TgDyrk1a and Ts65Dn	male	3 months	Mega Green Tea Extract (45% EGCG, 2–3 mg/day EGCG)	1 month	• Rescued object recognition memory impairment	^20^
mBACtgDyrk1a and Ts65Dn	male	3–4 months	Polyphenon 60 (27% EGCG, 60 mg/kg/day EGCG) Mega Green Tea Extract (45% EGCG, 60 mg/kg/day EGCG)	4–6 weeks	• Improved novel object recognition discrimination in YACtgDyrk1a mice • Improved spontaneous alternation in Ts65Dn mice	^60^
TgBACDyrk1A	male	2 months	FontUp (0.5% EGCG, 5 mg/kg/day EGCG)	2–8 days	• Increased plasma homocysteine • Increased glutathione • Decreased alanine aminotransferase • Increased BDNF • Normalization of erk and Akt phosphorylation	^31^

In contrast to the studies in Table 1, experiments in our laboratory that administered pure EGCG to Ts65Dn mice found no significant improvement in DS cognitive or behavioral phenotypes. In those studies, EGCG was administered in the drinking water beginning in early adolescence in dosages of ~9, 20, or 50 mg/kg/day^22,23^. The multiple, substantial differences in the methods and types of EGCG administration in different experimental mouse models make it difficult to reconcile outcome differences by direct comparison across studies. One important factor that cannot be ruled out is that polyphenol mixtures of green tea extracts contain other catechins with bioactive potential [epigallocatechin (EGC), epicatechin-3-gallate (ECG), and epicatechin (EC)] that are not present in pure EGCG treatments, and high plasma levels of these other catechins have been reported after consumption of green tea extract^24,25^. Another key factor is that the daily dosage of EGCG also varied across the mouse model studies. In studies using green tea extract (in drinking water or chow), daily EGCG consumption has ranged from ~20 to 200 mg/kg/day. In the pure EGCG studies, the daily dosage was either ~9, 20, or 50 mg/kg/day, raising the possibility that the lack of effects of pure EGCG in DS mouse models may be due to lower daily dosages. In most studies, the daily amount and temporal distribution of EGCG/green tea extract was controlled by the consumption pattern of the mouse rather than by the experimenter. Levels of EGCG in blood or tissue produced by the treatments have rarely been reported, further hindering comparisons of EGCG effects across studies. The bioavailability of ECGC or other bioactive green tea polyphenols is quite low in rodents (4.9 to 6.7%) after oral administration and varies with route of administration and species^26–29^. These considerations highlight the need to determine levels of EGCG that are produced in preclinical DS models of EGCG treatment.

To begin to address these gaps, the current study delivered EGCG in dosages of 200 mg/kg/day via oral gavage. This dose was chosen to achieve a high daily exposure within the range of EGCG doses reported to be given to individuals with DS by their caregivers^30^ and that approximated the highest daily dosage reported for the preclinical studies (non-DS) with green tea extract (in drinking water). Gavage EGCG treatment with 217 mg/kg/day for two weeks did not produce tissue histopathology in mice^27^. The current study first determined serum levels of EGCG after acute treatment. Then, cognitive and skeletal DS phenotypes were assessed during a 3-week gavage treatment. Four main hypotheses were tested: (1) that acute doses of 200 mg/kg EGCG will produce measurable blood levels of EGCG in trisomic mice; (2) that daily oral gavage treatments will improve cognitive and skeletal phenotypes in the trisomic mice; (3) that the DS mice will have elevated expression of DYRK1A protein in brain; and (4) that DYRK1A-associated kinase activity in brain and bone will be reduced by EGCG.

## Results

### Serum concentrations of EGCG following acute gavage treatment

**Figure 1:**
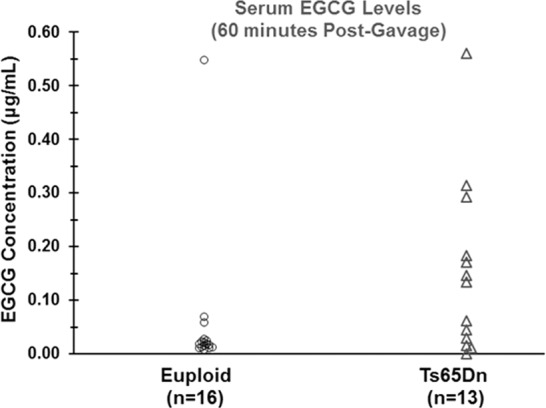
Serum concentration of Epigallocatechin-3-gallate (EGCG) 60 minutes after gavage treatment with 200 mg/kg EGCG in male Ts65Dn mice and euploid littermates. EGCG levels were determined by liquid chromatography and tandem mass spectroscopy (LC-MS/MS) using standards ranging between 0.001 and 0. 06 µg/mL. All samples yielded a non-zero value, and the Ts65Dn mice had significantly higher EGCG levels than the euploid mice (p = 0.022, Mann-Whitney U).

The concentrations of EGCG (µg/ml) in the serum after acute dosage of 200 mg/kg EGCG treatment for euploid and trisomic mice at 30, 60, and 90 minutes post-gavage are shown in Table 2. The majority of serum samples were collected at 60 minutes; the others were randomly sampled at either 30 or 90 minutes. Non-zero levels of EGCG were detected in all mice. As shown in Fig. 1, at 60 minutes the distributions for euploid and trisomic groups differed significantly (U = 155.5, p = 0.022, n = 29), with trisomic mice having higher ECCG concentrations (*Mdn* = 0.134 µg/ml [0.292 µM]; mean rank =18.96) than euploid mice (Mdn = 0.018 µg/ml [0.039 µM]; mean rank=11.78). Serum concentration mean ranks across the three time points did not differ significantly for either genotype (Kruskal-Wallis test, p’s>0.20).

**Table 2:** Median (and interquartile range) of serum EGCG concentrations (µg/ml) at 30, 60, and 90 minutes after gavage treatment with 200 mg/kg EGCG.

Group	Time Post-Gavage (minutes)		
	30	60	90
Euploid	0.036 (0.024–0.060) n = 5	0.018 (0.011–0.026) n = 16	0.027 (0.014–0.076) n = 8
Ts65Dn	0.052 (0.020–0.224) n = 4	0.134^a^ (0.022–0.238) n = 13	0.028 (0.018–0.282) n = 5

^a^Significantly different from Euploid 60-minute group (p = 0.022, Mann-Whitney U).

### Body weight with chronic treatment

**Figure 2:**
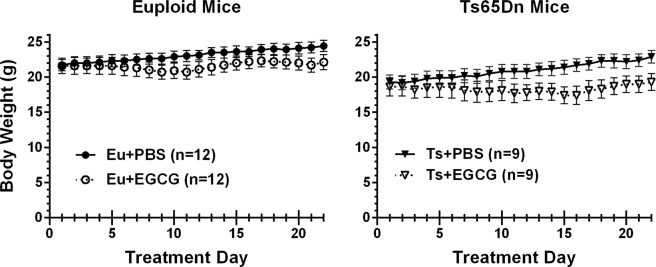
Body weights of male Ts65Dn and euploid littermates over the 3-week course of gavage treatment. Littermates were randomly assigned to either PBS or EGCG daily gavage treatments beginning on PD 42. EGCG treatment resulted in significant growth restriction in both genotypes that emerged over the 3-week treatment period.

As expected, Ts65Dn mice weighed significantly less than euploid mice at the beginning of treatment (18.9 + 0.8 g and 21.6 + 0.7 g, respectively; see Fig. 2) and the group difference was evident throughout the 3-week treatment period [main effect of genotype, F(1,38) = 7.322, p = 0.010]. Daily EGCG gavage resulted in significant growth restriction over weeks compared to PBS gavage in both the euploid and the Ts65Dn groups, with differences between PBS and EGCG groups increasing over time [treatment × day interaction, F(3.19,121.22) = 9.874, p < 0.001; main effect of treatment, F(1,38) = 4.126, p = 0.049; main effect of day, F(21,798) = 18.475, p < 0.001]. Despite the trend for larger effects of EGCG on the weights of Ts65Dn mice as treatment progressed, no treatment × genotype interactions were statistically significant.

### Multivariate concentric square field (MCSF)

**Figure 3:**
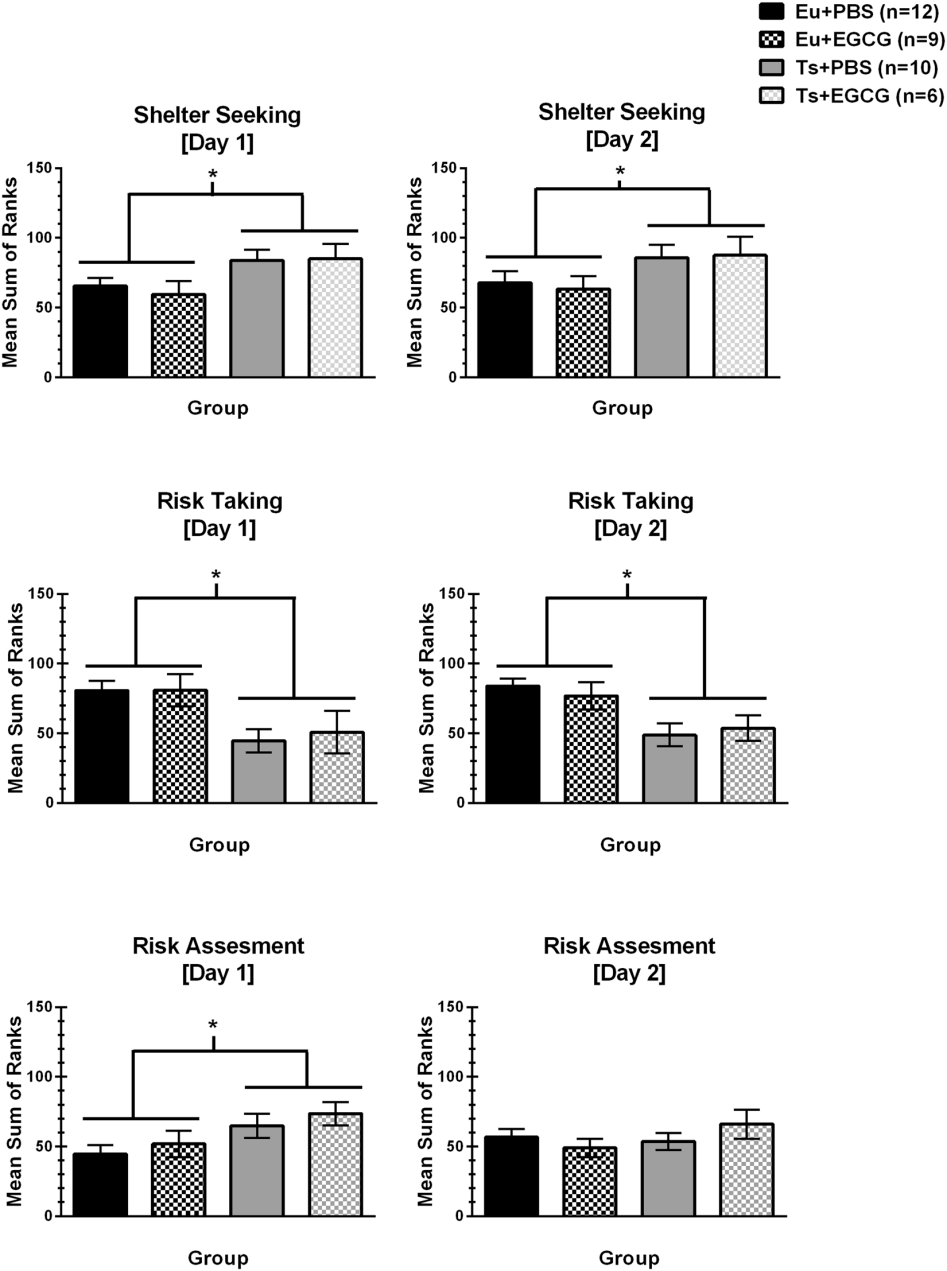
Multivariate Concentric Square Field (MCSF). Significant differences were seen between trisomic and euploid mice for shelter seeking, risk taking and risk assessment. No effect of EGCG treatment was seen in Ts65Dn or littermate control mice.

There were no significant group differences in locomotor activity (total number of zone entries) or in the category of exploratory behavior on either Day 1 or Day 2, indicating that neither genotype nor EGCG treatment significantly affected overall activity or exploration of novel features of the MCSF. In addition, there were no main or interactive effects of EGCG treatment on any of the five behavioral categories. Trisomic mice differed from euploid littermates on the other three categories of behavior (shelter seeking, risk assessment, risk taking), shown in Fig. 3.

On the first day of MCSF testing (left panels), the trisomic mice as compared to euploid mice engaged in significantly more shelter seeking [main effect of genotype, F(1,32)=7.126, p = 0.012], significantly less risk taking behavior [main effect of genotype, F(1,33)=10.786, p = 0.002], and significantly more risk assessment [main effect of genotype, F(1,33)=6.043, p = 0.019]. Similarly on Day 2, trisomic mice again displayed significantly more shelter seeking [main effect of genotype, F(1,33)=4.415, p = 0.043] and significantly less risk taking [main effect of genotype, F(1,33)=6.043, p = 0.019], but no longer differed significantly in risk assessment. Overall, these outcomes suggest that the trisomic mice are more risk averse and engage more in shelter seeking than euploid mice in the complex novel environment of the MCSF, and EGCG treatment did not affect these phenotypes.

### Balance beam

The mean (±SEM) number of paw slips at each of the three beam widths are shown in Table 3. All mice exhibited an increase in paw slips as the widths decreased (main effect of beam width, F(1,80) = 86.92, p ≤ 0.001), but there were no group differences at any beam width as (no main or interactive effects of genotype or treatment).

**Table 3:** Balance Beam Performance. Mean (± SEM) number of paw slips at each beam width.

			Beam Width (mm)^a^		
Group	Treatment	n	12	9	6
Euploid	PBS	13	2.4 ± 0.3	4.6 ± 0.5	6.4 ± 0.7
Euploid	ECGG	11	2.0 ± 0.5	4.7 ± 0.7	5.1 ± 1.2
Ts65Dn	PBS	12	3.1 ± 0.6	4.7 ± 0.7	6.3 ± 0.6
Ts65Dn	EGCG	8	2.1 ± 0.4	4.8 ± 0.6	7.2 ± 1.0

^a^Main effect of beam width (p < 0.001).

### Morris Water Maze (MWM)

#### Acquisition

**Figure 4:**
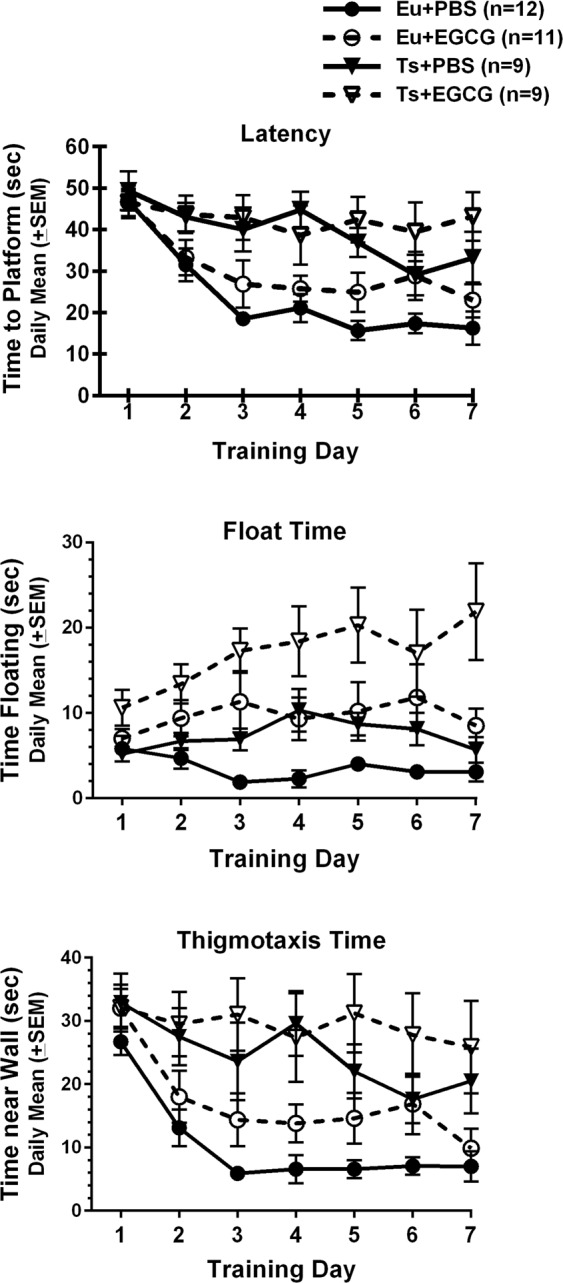
Morris water maze acquisition. [Top panel]: Mean (± SEM) latencies (on four trials per session) to reach the hidden platform over the seven days of acquisition training. Acquisition deficits in the trisomic mice were confirmed by the significantly longer escape latencies as compared to euploid littermates, and EGCG treatment failed to improve those deficits. [Middle panel]: Mean (± SEM) time spent floating (on the four trials per session) over the seven days of acquisition training. EGCG treatment produced significant increases in floating behavior over the training period, and the Ts65Dn mice floated more than euploid littermates. [Bottom panel]: Mean (± SEM) time spent within 25 cm of the tank wall (thigmotaxis) over acquisition training. Euploid mice showed significantly greater reduction in thigmotaxis over days than trisomic mice.

The trisomic mice showed the expected deficits in acquisition of place learning. Mean daily latencies to find the platform (Fig. 4, panel A) declined significantly across the seven days of training [main effect of day, F(6,222) = 14.18, p < 0.001], but the euploid mice showed significantly greater reductions in mean daily latencies than did the trisomic mice [genotype × day interaction, F(6,222) = 3.746, p = 0.001; main effect of genotype, F(1,37) = 20.217, p < 0.001]. EGCG treatment did not significantly affect escape latencies. Path lengths (Table 4) also significantly declined over days for all groups [main effect of day, F(6,222) = 27.935, p < 0.001], and the trisomic mice had significantly longer mean path lengths [main effect of genotype F(1,37) = 16.648, p < 0.001]. There were no significant main or interactive effects of EGCG treatment on path length. EGCG treatment significantly increased floating behavior (Fig. 4, middle panel) in both the trisomic and euploid mice [main effect of EGCG: F(1,37) = 17.597, p < 0.001]. In addition, trisomic mice as compared to euploid littermates spent significantly more time floating, main effect of genotype [F(1,37) = 8.893, p = 0.005]. The trend for increasing time floating over days in the EGCG-treated trisomic mice did not yield statistically significant interactive effects of day. However, the increased floating over days in EGCG-treated groups was associated with significant reductions in swimming speed in the EGCG-treated groups of both genotypes (Table 4) that emerged across days [treatment × day interaction, F(6,222) = 2.878, p = 0.01; main effect of treatment, F(1,37) = 9.016, p = 0.005; main effect of day, F(6,222) = 11.057, p < 0.001]. The acquisition deficits of the trisomic mice were also associated with significantly more thigmotaxic behavior (time spent within 25 cm of the wall) compared to euploid mice (Fig. 4, Panel C). Thigmotaxis generally declined over the course of acquisition (main effect of day; F(6,222) = 13.288, p < 0/001), but the trisomic mice showed significantly less reduction in thigmotaxis over days than euploid mice [day × genotype interaction, F(6,222) = 3.506, p = 0.007; main effect of genotype, F(1,37) = 16.248, p < 0.001].

**Table 4:** Additional performance measures during acquisition training in the Morris water maze. Eu=euploid; Ts=trisomic; PBS/EGCG = chronic gavage treatment.

Measure	Group	MWM Acquisition Training Day						
		Day 1	Day 2	Day 3	Day 4	Day 5	Day 6	Day 7
Path Length (meters) Mean ± (SEM)^a.b^	**Eu** + **PBS** (n = 12)	8.95 (0.53)	6.33 (0.72)	3.89 (0.37)	4.20 (0.59)	2.70 (0.52)	3.43 (0.48)	2.84 (0.75)
	**Eu** + **EGCG** (n = 11)	9.12 (0.99)	5.40 (0.79)	3.50 (0.60)	3.55 (0.59)	3.13 (0.48)	3.36 (0.64)	3.34 (0.57)
	**Ts** + **PBS** (n = 9)	9.69 (1.14)	8.17 (0.82)	7.13 (1.02)	7.16 (0.95)	5.75 (0.77)	4.26 (1.03)	5.69 (1.34)
	**Ts** + **EGCG** (n = 9)	8.20 (1.16)	7.16 (0.57)	5.41 (0.93)	4.03 (0.65)	4.62 (0.65)	4.99 (0.94)	4.62 (0.63)
Speed (m/sec) Mean ± (SEM)^c,d,e^	**Eu** + **PBS** (n = 12)	0.190 (0.008)	0.202 (0.010)	0.200 (0.008)	0.204 (0.006)	0.143 (0.015)	0.179 (0.004)	0.183 (0.012)
	**Eu** + **EGCG** (n = 11)	0.185 (0.010)	0.158 (0.012)	0.143 (0.016)	0.145 (0.017)	0.140 (0.015)	0.146 (0.017)	0.143 (0.014)
	**Ts** + **PBS** (n = 9)	0.189 (0.010)	0.189 (0.010)	0.168 (0.008)	0.156 (0.014)	0.149 (0.013)	0.140 (0.015)	0.156 (0.012)
	**Ts** + **EGCG** (n = 9)	0.175 (0.016)	0.160 (0.016)	0.128 (0.014)	0.121 (0.014)	0.121 (0.018)	0.143 (0.020)	0.121 (0.019)

^a^Main effect of day. ^b^Main effect of gentoype. ^c^Main effect of treatment.

^d^Main effect of day. ^e^Interactive effect of treatment × day.

#### Probe trial

**Figure 5:**
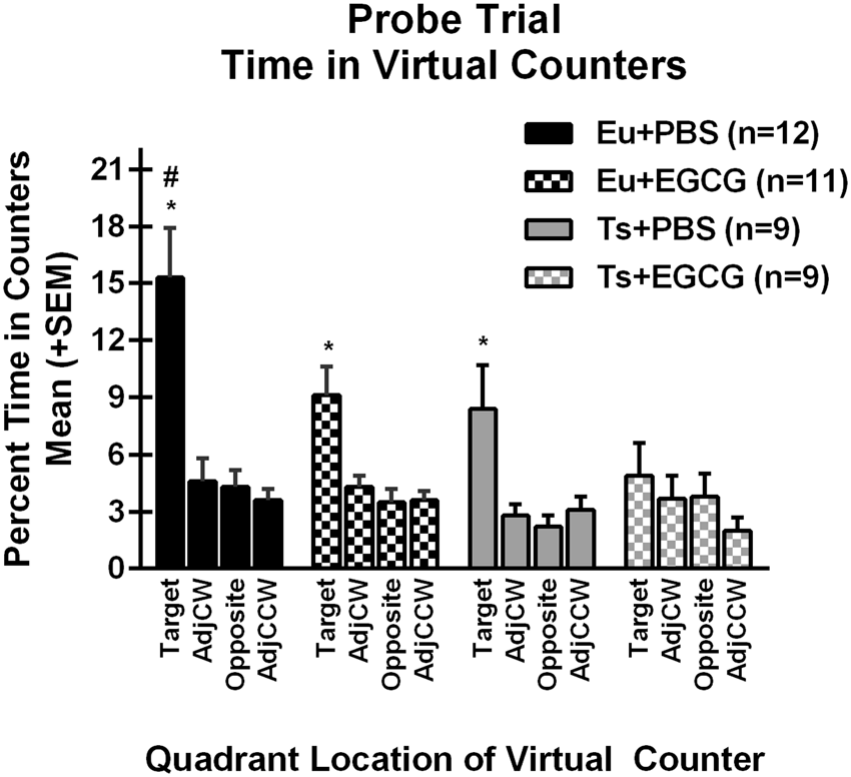
Morris water maze probe trial search. For each of the four groups, the percent of time (of the 60-second probe trial) that was spent in each of the four virtual counters are shown as function of quadrant relative to the location of the target quadrant (Target, Adjacent-clockwise, Opposite, Adjacent-counterclockwise). Note the significant reduction in target search bias in the trisomic mice, and the reduction in target search bias in the groups given EGCG. *Significantly greater than other three quadrants for the same group; ^#^Significantly greater than the time in Target of all other groups.

A repeated measures ANOVA on percent time in in the four quadrant zones on the probe trial yielded the expected overall main effect of quadrant, F(3,111) = 22.949, p < 0.001, with the generally greater percent time in the Target counter consistent with the expression of spatial memory (Fig. 5). The euploid mice given PBS spent significantly more time in the Target zone than any of the other three groups [genotype × quadrant interaction, F(3,111) = 3.235, p = 0.025; main effect of genotype, F(1,37) = 9.060, p = 0.005; protected LSD post hoc tests on time in Target, p < 0.05], consistent with stronger spatial search strategies for the euploid-PBS group. Unexpectedly, treatment with EGCG significantly disrupted the expression of place biases in the probe trial [treatment × quadrant interaction, F(3,111) = 3.940, p = 0.01] and the EGCG-treated euploid group spent significantly less time in the Target counter than the euploid-PBS group. Notably, the trisomic mice given EGCG failed to express a significant place bias the probe trial. The probe trial deficits in search biases in the trisomic groups were associated with alterations in other performance measures (see Table 5). These included reduced probe trial path length in trisomic mice [main effect of genotype, F(1,37) = 10.009, p = 0.003], slower swimming speed [main effect of genotype, F(1,37) = 9.732, p = 0.004], increased thigmotaxis time [main effect of genotype, F(1,37) = 11.495, p = 0.002], and increased time floating [main effect of genotype, F(1,37) = 9.525, p = 0.004]. In addition, the adverse effects of EGCG treatment on probe performance were evident in EGCG-induced increased thigmotaxis time [main effect of treatment, F(1,37) = 4.969, p = 0.032] and increased time floating [main effect of treatment, F(1,37) = 4.634, p = 0.038].

**Table 5:** MWM Probe trial additional performance measures.

Group	Path Length (meters)^a^	Speed (m/sec)^a^	Thigmotaxis (sec)^a.b^	Float Time (sec)^a.b^
Eu + PBS (n = 12)	13.3 (0.6)	0.222 (0.009)	16.1 (2.7)	4.0 (1.7)
Eu + EGCG (n = 11)	12.0 (0.8)	0.200 (0.014)	24.7 (3.5)	7.8 (2.4)
Ts + PBS (n = 9)	10.4 (0.8)	0.174 (0.012)	29.0 (4.5)	10.7 (3.6)
Ts + EGCG (n = 9)	9.1 (1.5)	0.152 (0.024)	36.6 (4.1)	20.2 (4.7)

Data for each group are shown as mean ± (SEM). Eu = euploid; Ts = trisomic; PBS/EGCG = chronic gavage treatment.

^**a**^Main effect of genotype.^ b^Main effect of treatment.

### Micro CT analysis of femora

Trabecular bone architecture measures in the distal femoral metaphysis were all impacted by trisomy (Table 6). Ts65Dn mice as compared to euploid mice exhibited significantly reduced bone mineral density, bone volume/total volume (BV/TV), trabecular thickness (Tb.Th), and trabecular number (Tb.N). Trabecular separation (Tb.Sp) was significantly greater in trisomic as compared to euploid mice. Treatment with 200 mg/kg/day EGCG had no significant impact on the trabecular bone.

**Table 6:** Skeletal structure in trabecular and cortical bone of femora from Ts65Dn and euploid mice given 200 mg/kg/day EGCG or PBS (Mean ± SEM).

Skeletal structure	Euploid + PBS (n = 12)	Euploid + EGCG (n = 12)	Ts65Dn + PBS (n = 11)	Ts65Dn + EGCG (n = 9)
Trabecular Bone				
Bone mineral density (BMD) (g/mm^3^)^a^	284 ± 12	248 ± 15	222 ± 11	208 ± 19
Percent bone volume (BV/TV) (%)^a^	31.4 ± 1.8	26.6 ± 2.0	22.1 ± 1.4	20.8 ± 2.4
Trabecular thickness (Tb.Th) (mm)^a^	0.079 ± 0.003	0.076 ± 0.003	0.071 ± 0.002	0.069 ± 0.002
Trabecular separation (Tb.S) (mm)^a^	0.165 ± 0.006	0.182 ± 0.011	0.201 ± 0.008	0.205 ± 0.009
Trabecular number (Tb.N) (1/mm)^a^	3.96 ± 0.17	3.50 ± 0.21	3.12 ± 0.21	3.00 ± 0.36
Cortical bone				
Total cross sectional area (CSA) (mm^2^)^a^	1.77 ± 0.05	1.84 ± 0.07	1.58 ± 0.04	1.50 ± 0.07
Marrow area (Ma.Ar) (mm^2^)^a,b^	0.76 ± 0.03	0.88 ± 0.03	0.62 ± 0.03	0.68 ± 0.02
Cortical area (Ct.A) (mm^2^)^a,b^	1.01 ± 0.03	0.96 ± 0.05	0.95 ± 0.02	0.81 ± 0.06
Cortical thickness (Ct.Th) (mm)^b^	0.26 ± 0.01	0.24 ± 0.01	0.26 ± 0.01	0.22 ± 0.01
Periosteal bone surface (P.Bs) (mm)^a^	5.56 ± 0.07	5.66 ± 0.10	5.34 ± 0.06	5.20 ± 0.13
Endocortical bone surface (Es.BS) (mm)^a,b^	3.84 ± 0.08	4.11 ± 0.06	3.56 ± 0.07	3.65 ± 0.06
Imax (mm^4^)^a^	0.30 ± 0.02	0.31 ± 0.03	0.27 ± 0.01	0.23 ± 0.03
Imin (mm^4^)^a^	0.14 ± 0.01	0.15 ± 0.01	0.11 ± 0.00	0.09 ± 0.01
Polar moment of inertia (J) (mm^4^)	0.44 ± 0.02	0.45 ± 0.04	0.37 ± 0.02	0.32 ± 0.04

^a^Main effect of genotype.

^b^Main effect of treatment.

Femurs from trisomic mice were smaller than euploid littermates, and treatment caused the femur to have a larger marrow cavity with a similar overall size leading to decreased cortical thickness and area. Trisomic as compared to euploid mice had reduced total cross-sectional area (CSA), marrow area (Ma.Ar), cortical area (Ct.Ar), periosteal bone surface (Ps.BS), endocortical bone surface (Es.BS), maximum moment of inertia (I_max_) and minimum moment of inertia (I_min_). Treatment with 200 mg/kg/day EGCG significantly decreased cortical thickness (Ct.Th) and Ct.Ar and increased Ma.Ar and endocortical bone surface (Es.BS) (Table 6).

In agreement with prevailing knowledge, trisomy negatively impacted the mechanical properties in the femur. The femurs of trisomic mice were significantly weaker than euploid mice, indicated by reduced ultimate force, stiffness, increased displacement to yield, yield stress, modulus, and resilience. These effects are likely attributed to the size of the femur rather than material properties. Treatment with 200 mg/kg EGCG decreased ultimate force and stiffness. There was a significant genotype × treatment interaction for ultimate stress. Despite having smaller bones (as reflected in decreased whole bone stiffness and strength), trisomic bones appeared to have better material properties (stiffer and stronger bone material). Treatment, if anything, made the bones less stiff and strong, potentially through its effects on bone size rather than material changes (Table 7).

**Table 7:** Mechanical data of femora from Ts65Dn and euploid mice given 200 mg/kg/day EGCG or PBS (Mean ± SEM).

Measure	Euploid+PBS (n = 11)	Euploid+EGCG (n = 9)	Ts65Dn+PBS (n = 11)	Ts65Dn+EGCG (n = 9)
Yield Force (N)	14.4 ± 1.1	11.8 ± 1.4	13.6 ± 0.6	11.7 ± 1.8
Ultimate Force (N)^a,b^	23.1 ± 1.0	20.6 ± 1.5	19.8 ± 0.6	17.0 ± 1.4
Displacement to Yield (µm)^a^	119.3 ± 7.7	107.9 ± 7.0	138.3 ± 11.3	142.3 ± 15.6
Postyield Displacement (µm)	533.0 ± 97.2	416.50 ± 96.2	384.8 ± 58.9	563.00 ± 170.5
Total Displacment (µm)	652.3 ± 97.5	524.4 ± 94.4	523.1 ± 67.0	705.3 ± 169.5
Stiffness (N/mm)^a,b^	137.4 ± 7.6	120.5 ± 8.2	112.5 ± 6.1	90.2 ± 8.0
Work to Yield (mJ)	0.97 ± 0.11	0.75 ± 0.14	1.08 ± 0.13	1.02 ± 0.26
Postyield Work (mJ)	9.8 ± 1.5	6.3 ± 1.3	6.8 ± 1.0	6.8 ± 0.9
Total Work (mJ)	10.8 ± 1.5	7.0 ± 1.3	7.9 ± 1.1	7.8 ± 1.0
Yield Stress (MPa)^a^	117.0 ± 8.1	93.3 ± 8.3	132.7 ± 8.2	128.6 ± 11.7
Ultimate Stress (MPa)^c^	187.8 ± 7.4	163.4 ± 5.4	191.9 ± 7.7	194.9 ± 4.8
Strain to Yield (me)	17.2 ± 1.1	15.9 ± 1.1	17.7 ± 1.4	17.9 ± 2.2
Total Strain (me)	94.0 ± 14.4	76.9 ± 14.0	67.0 ± 8.5	85.8 ± 18.8
Modulus (GPa)^a^	7.78 ± 0.45	6.58 ± 0.28	8.49 ± 0.44	8.38 ± 0.47
Resilience (MPa)^a^	1.13 ± 0.12	0.87 ± 0.14	1.36 ± 0.19	1.39 ± 0.30
Toughness (MPa)	12.7 ± 1.8	8.3 ± 1.4	10.1 ± 1.6	11.8 ± 2.1

^a^Main effect of genotype.

^b^Main effect of treatment.

^c^Genotype × treatment interaction.

Abbreviations: N: Newtons; mJ: millijoules; MPa: megapascal; me: microstrain; GPa: gigapascal.

### DYRK1A protein levels

**Figure 6:**
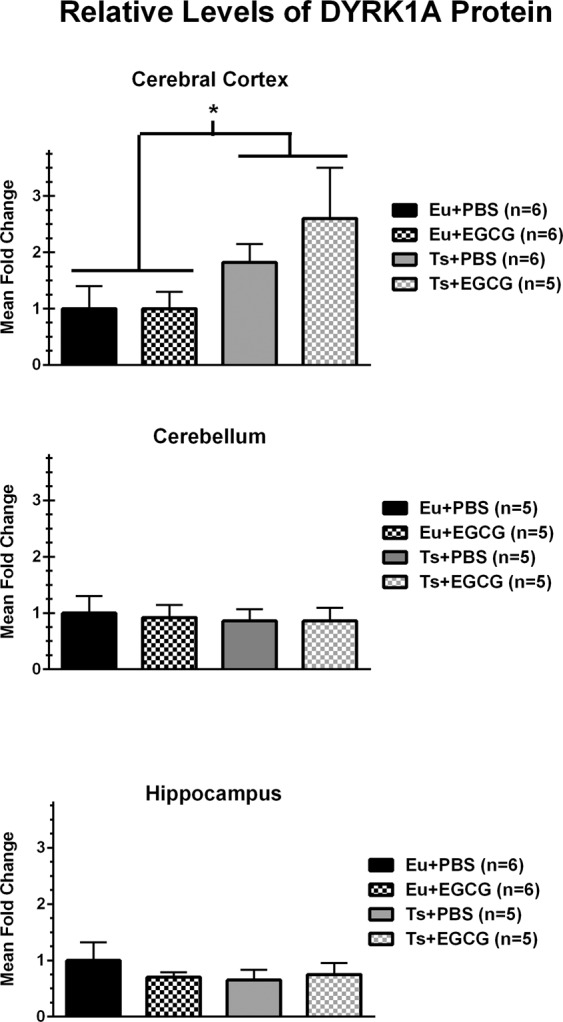
Western blot analyses of relative DYRK1A protein levels for cerebral cortex, hippocampus, and cerebellum in 68-day-old euploid and trisomic male mice. DYRK1A from each brain region was normalized to actin as a loading control, then each sample ratio was expressed as a relative proportion of the mean value of the euploid-PBS control group for that brain region. DYRK1A was significantly overexpressed in trisomic mice only in the cerebral cortex (main effect of genotype, p = 0.034).

As shown in Fig. 6, for the cerebral cortex (top panel) the trisomic mice had significantly higher relative DYRK1A levels than euploid mice [main effect of genotype, F(1,19)=5.230, p = 0.034], with a 2.1-fold average increase relative to euploid mice (collapsed across treatment). There were no significant differences in DYRK1A protein levels between genotypes in the hippocampus and cerebellum, and EGCG treatment did not significantly affect DYRK1A protein levels in any region.

### Kinase activity

**Figure 7:**
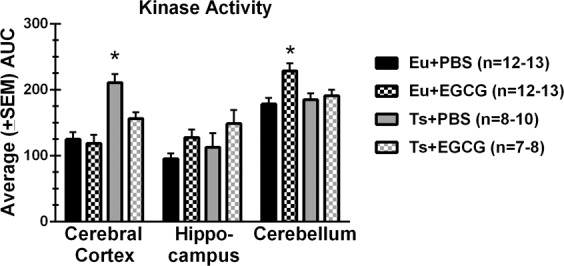
DYRK1A-related kinase activity in brain regions of Ts65Dn and control mice with and without EGCG treatment. The area under the curve (AUC) of the the p-FAM-Woodtide fluorescence was measured from protein isolated from brain regions of treated and untreated mice. DYRK1A-related kinase activity was significantly higher in the cerebellum than the other brain areas. A reduction in kinase activity in trisomic as compared to euploid mice was only found in the cerebral cortex. ECGC treatment increased kinase activity in the hippocampus and cerebellum, with kinase activity in Ts65Dn + EGCG treatment significantly higher than Ts65Dn + PBS treated animals.

As shown in Fig. 7, kinase activity was significantly higher in the cerebellum than in the cortex or hippocampus [main effect of region, F(2,55.8)=36.88, p < 0.001], and differences between trisomic and euploid mice depended on region [region × genotype interaction, F(1.59,55.81)= 7.899, p < 0.001; main effect of genotype, F(1,35)=9.696, p = 0.004]. Moreover, the effects of EGCG treatment depended on brain region [region × treatment interaction, F(1.59,55.81)= 5.002, p < 0.001]. In the cerebral cortex, follow-up two-way ANOVA confirmed significant effects of genotype [F(1,40)=28.59, p < 0.001] and EGCG treatment [F(1,40)=7.01, p = 0.012]. Post hoc LSD tests indicated that the PBS-treated trisomic mice had higher kinase activity than PBS-treated and EGCG-treated euploid groups (p < 0.001 and p = 0.019, respectively), and ECGC significantly reduced kinase activity in the trisomic mice compared PBS-treated trisomic mice (p = 0.005). In contrast, kinase activity in the cerebellum and hippocampus did not differ significantly between trisomic and euploid groups. EGCG treatment produced a significant and unexpected increase in kinase activity both in the cerebellum [main effect of treatment, F(1,37)=8.314, p = 0.007] and the hippocampus [main effect of treatment, F(1,38)=5.332, p = 0.026]. Post hoc LSD tests indicated that the ECGG-induced increase in kinase activity in the cerebellum was significant only in the euploid group (p = 0.001), but in the hippocampus group differences within each genotype did not reach significance.

For the femur, there were no statistically significant effects of genotype or treatment on kinase activity (Euploid + PBS:117.8 ± 13.3, n = 13; Euploid+EGCG: 97.0 ± 19.5, n = 13; Trisomic+PBS: 95.0 ± 13.5, n = 7; Trisomic+EGCG: 76.3 ± 16.4, n = 10).

## Discussion

The first step in understanding the discrepant outcomes across studies using treatments of EGCG or green tea extracts containing EGCG in DS mouse models is to identify the *in vivo* levels of EGCG produced by different treatments. This study documents serum levels of EGCG in a mouse model of DS following controlled daily dosing of 200 mg/kg pure EGCG using oral gavage, confirming our hypothesis that this dose and route of EGCG treatment produced measurable levels of EGCG in the blood in Ts65Dn and euploid mice. Other experiments with considerably less EGCG in combination with other components have also shown measurable EGCG levels in the brain and bone of TgDyrk1A mice^31^. This experimenter-controlled gavage dosing contrasts with most other studies administering EGCG in DS mouse models that typically provide EGCG (or green tea extracts containing EGCG and other catechins) in the drinking water, in which the daily dosage and pattern of EGCG (or green tea extract) consumed over the day is controlled by the drinking behavior of the subject rather than directly by the experimenter. In normosomic Swiss albino mice, a single oral gavage EGCG dose of 108 mg/kg produced peak EGCG levels of ~0.13 µg/ml^27^. By comparison, the daily gavage administration of 200 mg/kg in the two-day protocol of the current study produced median EGCG levels of 0.018 µg/ml in the serum of euploid mice one hour after the gavage on the second day, approximately 14% of the levels reported for the Swiss albino mice. Notably, the median serum EGCG level in trisomic mice in this study was 0.098 µg/ml, significantly higher than the euploid levels but closer to the peak level at 60 minutes when 108 mg/kg EGCG was given to Swiss albino mice^27^. The differences in blood levels of EGCG between the two studies may have arisen from any of multiple procedural differences. Ramachandran *et al*. used female Swiss albino mice, administered a single dose of EGCG using 4% DMSO and PBS as a vehicle, and performed multiple blood draws (without specifying whether serum or plasma was used), whereas the current study used male Ts65Dn mice on an ~50% B6 50% C3H background, administered EGCG in PBS without the use of DMSO once a day for two days, and obtained blood samples only once per animal via terminal cardiac puncture to measure serum EGCG. DMSO was likely used in the Ramachandran study to achieve the higher concentrations of treatment; the current study avoided DMSO because of its inhibitory effects on enzymatic activity^32–34^. The Ramachandran *et al*. study also used the divalent cation chelator EDTA as an anticoagulant for plasma/serum collection; EDTA can interfere with enzymatic activities that metabolize EGCG that may alter the temporal profile of EGCG elimination^35^. Conversely, our method of deproteinating the serum samples using acetonitrile may have precipitated proteins bound to EGCG, resulting in a reduced serum concentration. We utilized LC-MS/MS that measures the quantities of specific ionized fragments of EGCG, because it is more specific and sensitive than the HPLC method that was used by Ramachandran *et al*.^36^. The higher serum EGCG concentrations of the trisomic compared to euploid mice after gavage treatment in this study may reflect differences in pharmacokinetic processes related to absorption, distribution, metabolism or elimination of EGCG, analyses that are beyond the scope of the current study. The short (two-day) duration of the acute gavage dosing provided only a snapshot of EGCG serum concentration and may not accurately model changes due to effects of sustained daily gavage treatment from PD 42 to PD 68 used in the chronic dosing in this study.

Variation in the *in vivo* effects of EGCG (or green tea extract) across different studies may result from multiple chemical or pharmacokinetic properties of EGCG, including chemical degradation of EGCG over time in solution, the low bioavailability of EGCG, or the potential for biotransformation in the digestive tract prior to absorption^37,38^. ECCG bioavailability using acute oral gavage of pure EGCG in Swiss albino mice was reported to be only about 6.7%, and gavage doses of 108 mg/kg yielded peak plasma levels (C_max_) of 0.14 µg/ml and an elimination half-life (t_½_) of 68.4 minutes^27^. Bioavailability may also change over time with consumption. The peak plasma ECGC levels resulting from a bolus intragastric challenge dose of 750 mg/kg EGCG in fasting naïve CF-1 mice was 1.3-fold higher than in a comparison group of mice that had consumed EGCG in the diet over the previous 2 weeks (C_max_ of 15.45 µg/ml as compared to 6.65 µg/ml, respectively). In normosomic A/J mice consuming either 0.1% or 0.6% green tea extract in the drinking water, plasma levels of ECGC also declined over the first week, stabilizing around 0.80 µg/ml and 200–250 µg/ml, respectively^39^. Given the potential for wide variation in the blood levels of EGCG that depend on route, formulation, and type of supplement treatment provided, identifying the blood levels of EGCG and other catechins produced by treatment with EGCG or green tea extracts in DS mouse models is an essential first step in determining the basis for differences in outcomes on DS phenotypes.

Although no formal histological measures of toxicity were determined in the mice of this study, the chronic daily gavage treatment with 200 mg/kg significantly encumbered growth in euploid and trisomic mice. This dosage was chosen because of the lack of improvement in behavioral phenotypes in trisomic mice after treatment in our previous studies with lower daily doses of pure EGCG (~9 to ~50 mg/kg/day) in the drinking water^22,23^ and reported high doses of EGCG consumed by individuals with DS^30^. The reduced body weights in the current study were similar to other studies where a high dosage of EGCG was given by oral gavage^27,40^. Nevertheless, the 200 mg/kg/day EGCG treatment in the present study did not cause attrition due to death or other major adverse effects; a previous study reported only mild toxicity when an oral EGCG dosage of about half of the present study was used^27^. EGCG administration via oral gavage has much lower bioavailability than intravenous or intraperitoneal treatments, and intraperitoneal treatments with 108 mg/kg/day EGCG caused mortality within 8 days^27^. When green tea extract including 48.4% EGCG was given by oral gavage to B6C3F1 mice (similar background as the mice in the current study) for 5 days a week for 14 weeks, hepatotoxicity and olfactory and respiratory epithelial necrosis were seen in mice given 250 and 500 mg/kg green tea extract (~121 and 242 mg/kg EGCG)^40^. A meta-analysis of animal studies determined an acceptable daily intake of only 4.6 mg/kg/day EGCG using a strict safety standard^41^. An analysis of body composition in individuals with DS who were given green tea extract with ~9 mg/kg/day EGCG for 12 months showed a trend in males for less body weight gain and lower BMI at 12 months and 6 months after treatment was completed^21,42^. In general, it is evident that chronic treatment with EGCG or green tea extract is not without risk, and differences in EGCG serum concentrations between euploid and trisomic mice after a similar EGCG treatment in this study warrant quantification of EGCG levels in individuals with and without Ts21. Treatments of EGCG in humans should monitor possible weight loss and toxicity, especially with long duration of treatment^27,41,43,44^.

Even with the relatively high dose of EGCG administered in this study to trisomic mice that produced pharmacologically relevant levels of EGCG in the blood, there were only limited behavioral and no skeletal improvements in Ts65Dn mice, mostly disconfirming our second hypothesis that this EGCG treatment would improve behavioral and skeletal deficits in DS mouse models. Similar to other studies, the current investigation confirmed that trisomic and euploid mice have significant behavioral differences^22,23^. The behavior in the MCSF showed that trisomic mice exhibited more shelter seeking and less risk taking on both days of testing, and EGCG treatment had no effect on these phenotypes. In contrast, our previous study using a lower concentration of EGCG in the drinking water did not find a significant effect of genotype on risk taking, but did find a significant genotype × treatment interaction in which EGCG decreased risk taking in trisomic mice but increased it in euploid mice^23^. The lower risk taking in trisomic mice in the current study compared to the lack of differences in trisomic and euploid control mice (drinking control fluid) in our previous study suggests that the daily stress of gavage may have differentially influenced the risk-taking behavior of trisomic and control mice, as measures of risk-taking and risk assessment have been related to stress and anxiety in other studies using the MCSF test^45–47^. For the risk assessment measure, trisomic mice showed increases as compared to euploid mice on the first evaluation day, similar to our previous study with the lower concentration with EGCG in the drinking water. In contrast, the increased shelter seeking of the trisomic mice in the current study was not found in the previous study with EGCG in the drinking water^23^, again suggesting a differential effect of gavage treatment.

As has been shown in our previous studies, trisomic mice were deficient in both the acquisition and probe trial phases of the MWM^22,23^. Trisomic mice had significantly less reduction in escape latencies over training and spent significantly less time in the target quadrant zone during the probe trial compared to euploid groups. These deficits in trisomic mice were associated with significantly increased amount of time near the wall (thigmotaxis) and increased time spent floating, as has been previously reported^48^. EGCG treatment did not improve these deficits in trisomic mice. In fact, the gavage EGCG treatment significantly increased float time and concurrently reduced swimming speed during acquisition in both trisomic and euploid mice. In the probe trial, trisomic mice showed decreased swimming speed along with increased time floating and increased thigmotaxis, and EGCG treatment interfered with the expression of place biases, reducing the time in the target zone and increasing thigmotaxis in both genotypes.

Our previous study administering 50 mg/kg/day EGCG in the drinking water^23^ found comparable deficits in water maze acquisition and probe trial performance in trisomic mice that were not improved by EGCG, but in that study the EGCG treatment did not interfere with the probe trial performance. The disruption of acquisition and probe trial performance with the gavage dosing of EGCG in the current study, including the overall increase in floating behavior and thigmotaxis, suggests that this high dose had adverse effects on performance of the water maze task. Time floating in the MWM is often not reported in behavioral studies of DS mouse models, and given the results of the present study, should be quantified to account for performance differences that may confound the typically-reported measures of learning, i.e., latency and path length measures for acquisition and target zone search biases for spatial memory. The present study did not include a cued (visible-platform) version of the water maze task, leaving open the question as to whether the increase in floating by EGCG-treated trisomic mice as training progressed is specific to the spatial task^48^. Overall, the enhanced deficits on the behavioral tests of mice given EGCG treatment over training may be due to interactive effects of increased stress of the gavage, though it appears that most of the adverse behavioral effects may be attributed to the high dose of EGCG.

In a previous study, administration of 9 mg/kg/day of pure EGCG in the drinking water significantly improved trabecular bone deficits observed in Ts65Dn mice^49^, but neither 50 mg/kg/day of EGCG^23^ nor 200 mg/kg/day of EGCG (current study) improved these trabecular bone deficits in Ts65Dn mice. To the contrary, these higher dosages of EGCG were detrimental to several bone parameters including cortical area and cortical thickness in both euploid and trisomic mice^22,23^. Low doses (<10 mg/kg/day) of EGCG-containing green tea supplements also were detrimental to cortical bone, although they did improve some trabecular parameters^37^. Treatments with 200 mg/kg EGCG decreased ultimate force, and to some extent, yield force. EGCG reduced stiffness in both trisomic and euploid mice, indicating that a high dosage of EGCG negatively affected the mechanical and material properties of all bones. A 12-month treatment of green tea extract containing ~9 mg/kg/day EGCG in humans with DS noted lowered bone mass, especially in females^21,42^. These and previous data noting adverse effects in bone properties after treatment with EGCG or green tea extracts containing EGCG^23,37^ indicate that monitoring of bone parameters during and after treatment is warranted.

The last two hypotheses tested in this study were whether DYRK1A protein levels were elevated in different regions of the brain in the adult stage, and whether 200 mg/kg EGCG treatment reduced kinase activity (of which DYRK1A was expected to contribute) in brain and bone at ~10 weeks of age. Significant DYRK1A overexpression in the brain of Ts65Dn mice as compared to euploid mice was evident only in the cerebral cortex, with no differences in the cerebellum or hippocampus. In a previous study of brain regions in ~10-week-old Ts65Dn and euploid mice^23^, EGCG treatment reduced the kinase activity in the cerebral cortex in trisomic, but not euploid mice. Conversely, EGCG treatment increased kinase activity in the cerebellum and hippocampus of the mice, with a specific increase in the cerebellum of euploid mice. These data suggest that DYRK1A is not elevated in the cerebellum or hippocampus at ~10 weeks of age in trisomic mice, and that EGCG does not affect DYRK1A activity in the brain.

Although EGCG has been shown to inhibit DYRK1A activity *in vitro* (using the Woodtide peptide)^8,50^, there is little evidence to directly support that EGCG inhibits DYRK1A activity *in vivo*, and perhaps more importantly, that EGCG is specific in limiting only DYRK1A activity. The varied results in the different brain regions and bone after EGCG treatment may reflect a more broad inhibitory mechanism of EGCG than just decreasing DYRK1A activity^6,7,51^. The results from these and other studies suggest that three copies of *Dyrk1a* lead to overexpression of DYRK1A protein in some but not all tissues and may be dependent on developmental time^52^, as DYRK1A levels in forebrain from Ts65Dn mice decreased from postnatal day 5 through day 35^53^. Increased DYRK1A levels and activity in this and other studies indicates that, although EGCG may not lower DYRK1A activity *in vivo*, trisomic DYRK1A may still be a viable target for skeletal and cognitive phenotypes associated with DS^18,51,52^.

## Conclusions

Studies describing behavioral improvements after administrations of green tea extracts report EGCG doses ranging from ~40 to ~200 mg/kg/day^12,20,54^. However, it is difficult to ascertain the independent contribution of pure EGCG in these reported behavioral improvements, given the multiple catechins with bioactive potential that are present in the green tea extract. With doses of ~9 to ~200 mg/kg/day of pure EGCG, Ts65Dn mice have largely failed to significantly improve behavioral and cognitive deficits^22,23^. It may be that the other components besides (or in combination with) EGCG included in the green tea extracts are having positive effects on learning and memory in Ts65Dn mice. Additionally, other effects of green tea polyphenols including EGCG besides altering DYRK1A activity may account for some of the beneficial effects see when green tea polyphenols are given to DS model mice^7^. Taken together, the results from four separate studies from our laboratory strongly suggest that pure EGCG does not improve cognitive and behavioral deficits related to Ts21 in DS mouse models. Pure EGCG has not proven to be an effective therapeutic treatment for improving DS-related cognitive deficits in Ts65Dn mice, and it may carry some dose-related risk for interfering with growth and skeletal integrity.

## Methods

### Animals

Ts(17^16^)65Dn (Ts65Dn—stock number 001924 from the Jackson Laboratories [Bar Harbor, ME]) females (~50% C57BL/6 J and ~50% C3H/HeJ background) were bred to B6C3F1 males (stock number 100010) and produced Ts65Dn and euploid progeny. New Ts65Dn females and B6C3F1 males were purchased from the Jackson Laboratories approximately every six months and introduced into the breeding colony. Only male mice were used for subsequent testing; Ts65Dn females were reserved for colony maintenance. Offspring were genotyped using the Ts65Dn breakpoint PCR^55^ and screened for the C3H-derived retinal degeneration mutation *Pde6b*^*rd1*^ that causes blindness within the first few weeks of life^56^ (Protocol 22421: Standard PCR Assay - Generic Pde6b, The Jackson Laboratory). Mice homozygous for *Pde6b*^*rd1*^ were excluded from the study. After weaning, male mice were group-housed in a reverse 12:12 light:dark cycle with white light off between 0700-1900 to accommodate behavioral testing during the dark phase of the circadian cycle. Experiments with animals were carried out in accordance with the NIH Guide for the Care and Use of Laboratory Animals and received prior IACUC approval from IUPUI (protocols SC213R and SC255R).

### Epigallocatechin-3-gallate (EGCG) treatment and serum levels

EGCG treatment was prepared fresh daily by dissolving EGCG (>95% purity, as determined by LC/MS analyses^37^) in phosphate buffered saline (PBS) at a concentration of 15 mg/mL. The prepared EGCG was administered via oral gavage (13.34 mL/kg body weight) once daily (200 mg/kg/day). Daily oral gavage of an equivalent volume of PBS (13.34 mL/kg body weight) was given as the vehicle control.

#### Acute treatment for serum level determination

For the determination of serum levels of EGCC, 6-week-old male Ts65Dn and euploid mice were treated with 200 mg/kg/day EGCG (based on the weight of each animal) once a day for two days and euthanized under isoflurane anesthesia followed by cervical dislocation at 30, 60, or 90 minutes after the second treatment. Blood was collected by a cardiac draw, centrifuged to isolate the serum, and the serum was snap frozen.

#### Analysis of serum EGCG levels

Serum was analyzed by liquid chromatography tandem mass spectroscopy (LC-MS/MS) to quantify the concentrations of EGCG^37^. EGCG concentration was analyzed using an Agilent 1200SL HPLC coupled with an Agilent 6520 quadrupole time-of-flight mass spectrometer (MS). Samples were separated using reverse phase chromatography with a Zorbax Eclipse Plus C18 column (2.1 × 50 mm, 1.8 µm particle size) operating at a temperature of 55 °C with a solvent ratio of 30% A (water with 0.1% formic acid) and 70% B (acetonitrile with 0.1% formic acid). Standards were made with serum from PBS-treated mice at 0, 0.001, 0.003, 0.005, 0.01 and 0.06 μg/mL. The area under the curve (AUC) from the abundance of fragments 124.7 m/z and 168.8 m/z were used for quantification.

#### Chronic treatment for DS phenotype assessment

For the long-term assessment of behavioral, biochemical, and structural outcomes, male Ts65Dn and euploid mice were treated once daily by gavage with either 200 mg/kg EGCG or PBS (based on the weight of each animal) beginning on postnatal day (PD) 42 for the duration of the study (PD 68). Behavioral testing began on PD 49 with gavage treatments administered 30 minutes before behavioral testing.

### Timeline for behavioral testing

The cognitive and behavioral tasks were administered as previously described^22,23^. Starting on PD 49, mice underwent two days of testing on the multivariate concentric square field maze (MCSF), followed by three consecutive days of balance beam testing (PD 51–53). After two rest days, the mice were trained on the Morris water maze (MWM) task from PD 56–63. On PD 68, the mice were euthanized under isoflurane followed by cervical dislocation. The cerebral cortex, cerebellum, and hippocampus were rapidly dissected upon sacrifice, snap frozen, and stored at −80 °C for subsequent protein extraction. The mouse carcasses were stored at 4 °C before femurs were extracted. After extraction, femurs were wrapped with gauze soaked in 1X PBS then frozen at −20 °C before microcomputed tomography (μCT) scanning and mechanical testing.

### Multivariate concentric square field maze (MCSF)

Mice were tested in the MCSF on PD 45 and 46. The MCSF apparatus is a complex multi-compartment novel environment with various distinct areas and structures that animals may explore, including compartments that are open, elevated, or sheltered and that have characteristic features such as a hurdle, a ramp, an aperture for nose poking, and a small dark enclosure^23^. For each session, the animal was placed in the center zone facing the wall with no openings; its activity was video recorded for each 20-minute session in dim light (15–20 lx) in all areas except for the dark corner room (1–2 lx) and the lighted bridge area (320 lx). The MCSF was thoroughly cleaned between testing sessions with 70% ethanol. Photocell counts of nose pokes and fecal boli were recorded after each 20-minute session by the experimenter. All sessions were scored by three independent observers blind to experimental treatment and genotype. Results were averaged to obtain the score value that was used for each animal. The correlation of scores across all measures between the three raters was r ≈ + 0.99. Behavioral measures were obtained using The Observer XT Version 8 software (Noldus Information Technology, Wageningen, The Netherlands), and included measures of frequency of entries, duration, duration per visit, and latency to initial visit for each defined location within the MCSF. Measurements of rearing were not available due to unreliable identification of defined rearing events in video playback.

### Balance beam (BB)

The balance beam task was administered as previously described^23^. Briefly, the balance beam task was conducted in red light over a 3-day period in which the first 2 days were allotted for training and the third day designated for testing. On the first of two training days, mice were trained to walk across a raised 19 mm wide wooden beam, starting at varying distances from the box. On the second day, mice were trained to cross the 12 mm beam three consecutive times without stopping. On the third day, mice were tested on three trials each of the 12-mm, 9-mm, and 6-mm beams. Trials were recorded using a Logitech camera positioned on the end of the beam opposite the goal box to capture hind-leg slips, defined as either hind-leg slipping off the top surface of the beam. The number of hind-leg slips during each recorded trial was scored by three independent scorers blind to the genotype and treatment.

### Morris water maze (MWM)

The MWM task was administered as previously described using a 125-cm diameter tank filled with 26 °C water made opaque with white tempera paint^23^. Briefly, this task was carried out over an eight-day period in which the first seven days consisted of acquisition trials in which an invisible platform (nine cm diameter) was submerged in the center of the same quadrant of the tank (designated as the target location). The eighth day was a probe day in which the platform was removed to quantify the spatial search behavior. On acquisition days, cohorts of 2–4 mice were tested in daily sessions, with each mouse given four trials (60 seconds maximum) to find the submerged escape platform, after which the mouse remained on the platform for 10 seconds. The mouse either found the platform within the 60 seconds or was moved to the platform by the experimenter after 60 seconds elapsed. Four different start locations (out of eight possible locations) were pseudo-randomly assigned for each trial within a given session (one start location per quadrant). All start locations were randomized over the 7-day acquisition period. Twenty-four hours after the last training day, mice were given a single probe trial, in which the platform was removed and each mouse was placed in a start location directly opposite of the quadrant that had contained the platform and was allowed to swim for 60 seconds. All days of testing were recorded with video tracking using HVS image software (HVS Image, Mountain View, CA, USA).

### Microcomputed tomography (μCT) imaging and analysis

Left femurs were extracted and stored in PBS-soaked gauze at −20 °C and thawed prior to scanning with a high-resolution μCT system (SkyScan 1172, Bruker microCT, Belgium). Scanning conditions were calibrated each day using two cylindrical hydroxyapatite phantoms (0.25 and 0.75 g/cm3 CaHA). The distal condyle to the 3^rd^ trochanter of each femur was scanned using 60 kV, 12 µm resolution, and Al 0.5 mm filter. Femurs were rewrapped with PBS-soaked gauze and stored at −20 °C for future mechanical testing. Femur scans were reconstructed, rotated, and analyzed using NRecon (SkyScan, Bruker microCT, Belgium), DataViewer, CTan (SkyScan, Bruker microCT, Belgium), and MatLab software (MathWorks, Inc. Natick, MA). Trabecular and cortical bone analyses were performed using a previously published protocol^37,57^. Bone mineral density (BMD) was calculated using the hydroxyapatite phantoms as a standard. Analysis for the cortical bone was performed on a standard site chosen as 7 transverse slices at 60% of the bone’s total length away from the proximal end of the distal growth plate. Trabecular bone analysis was performed on the region of the distal metaphysis defined as 10% of total bone length, starting at the proximal end of the distal growth plate. The region of interest within the metaphyseal region was auto-segmented to exclude outer cortical bone using a custom Matlab code^37,57^.

### Mechanical testing

The mechanical properties of the femora were determined by 3-point bending (ElectroForce 3200; Eden Prairie, MN USA). The left femora were thawed to room temperature and tested in the AP direction (anterior surface in tension). The loading span was set at 6 mm and a preload of 0.5 N applied to the midpoint of the bone to establish contact. Once preloaded, the bone was monotonically tested to failure at a displacement rate of 0.025 mm/sec.

### High performance liquid chromatography (HPLC) kinase activity assay

Isolated protein was quantified by Bradford assay and subjected to a HPLC-based kinase activity assay as previously described^23^. Briefly, ~100 µg protein was incubated at 37 °C with a 5-carboxyfluorescein tagged Woodtide peptide (2 mg/mL) and kinase buffer. The addition of ATP (1 mM) started the reaction and samples were incubated at 37 °C for 30 min. The reaction was stopped by the addition of HClO_4_ (15%) and centrifuged at 4 °C for 10 min at 14,655 *g*. The supernatant was analyzed on the Agilent 1260 HPLC. Separation was performed using initial conditions of 85% H_2_O with 0.1% formic acid (solvent A) and 15% acetonitrile with 0.1% formic acid (solvent B) held for 1 min, followed by a stepwise gradient ending with 5% solvent A and 95% solvent B over 11 min. FAM-Woodtide fluorescence was measured using a fluorescence detector with an excitation wavelength of 485 nm and an emission of 530 nm. The peak height and retention time determined the area under the curve for the p-FAM-Woodtide and FAM-Woodtide. Results were analyzed using OpenLab CDS Chemstation software.

### Western Blot quantification of DYRK1A protein

Isolated protein lysates (20 μg) from the cerebral cortex, hippocampus, and cerebellum were resolved by electrophoresis on polyacrylamide gels (Bolt 4–12% Bis Tris Plus Gels), then transferred to PVDF membranes. Membranes were blocked in 5% milk in Tris Buffered Saline with 0.1% Tween 20 (TBS-T), incubated overnight at 4 °C in primary antibodies diluted in 5% milk-TBS-T as follows: rabbit anti-DYRK1A antibody, 1:500 (A303–802A, Bethyl Laboratories); mouse anti-beta-actin, 1:5000 (A2228, Sigma Aldrich), and labeled with donkey anti-rabbit IgG AlexaFluor 790 and donkey anti-mouse IgG AlexaFluor 680 secondary antibodies (1:10,000, Jackson Immunoresearch). Fluorescence was detected using a LI-COR CLx Imager. Each membrane included protein samples from each of the three brain regions from four different mice, one from each genotype and treatment combination. DYRK1A for each sample was normalized to actin and each normalized ratio was expressed as a proportion relative to the mean of the euploid-PBS group for the particular brain region.

### Data analyses

#### EGCG levels and body weight

The data for EGCG serum levels at 30, 60, and 90 minutes following acute gavage were not normally distributed (Shapiro-Wilk’s test, p < 0.001) so they were analyzed with non-parametric rank-order statistics. Samples from 51 mice were included in the serum EGCG analysis (see Table 2 for group numbers); values of one trisomic mouse and one euploid mouse were far beyond the assay distribution (> 10 standard deviations above the mean) and were excluded from the analysis. Group differences between euploid and trisomic mice at each time point were analyzed using Mann-Whitney U tests; comparison of effects at different times within each genotype used a one-way Kruskal-Wallis test. Body weights over the chronic treatment period were analyzed with a repeated-measures ANOVA using the Greenhouse-Geisser correction for violations of the assumption of sphericity.

#### Multivariate Concentric Square Field

The mean of each of the individual measures (e.g., latency to initial visit, duration, frequency, and duration per visit) was generated from the three independent observer scores, and scores for each of the five behavioral categories were tabulated as shown in Table 1 of our previous study^23^. Data from 13 subjects were unavailable for the analysis due to technical problems with the video recording of a session that precluded reliable scoring. The subjects that were unavailable were distributed as follows: one trisomic mouse given PBS, three trisomic mice given EGCG, five euploid mice given PBS, and four euploid mice given EGCG); one other animal was lethargic on the test day so was not tested (trisomic mouse given EGCG).

For the remaining mice, the total entries data (summed across the seven MCSF zones) constituted an underlying continuous variable and was distributed normally (Shapiro-Wilk’s W test), so those sums were analyzed separately for each day with a two-way factorial ANOVA with genotype and treatment as grouping factors. The other four behavioral categories (risk-taking, risk assessment, shelter seeking and exploratory behavior) involved measures that used different scales (time or frequency). As described in our previous study [20], the data for each contributing measure were first rank ordered for a given day, then the ranks for each measure contributing to a category were summed across the measures for each animal for that day to provide a single summed score for the animal for each category. The summed scores of subject ranks for each of these four categories formed a continuous underlying variable for each category and were normally distributed within each group in all but two cases (Shapiro-Wilk’s W test). The summed rank data for each category for a given day were analyzed (separately for each day) with a two-way factorial ANOVA with genotype and treatment as grouping factors.

#### Balance beam

The number of paw slips on the balance beam was analyzed using a mixed ANOVA with treatment group and genotype as between-group factors, and beam width (12 mm, 9 mm and 6 mm) as a repeated measure.

#### Morris water maze

The average daily latency, path length, time (sec) spent within 25 cm of the tank wall (thigmotaxis time), time spent floating, and swimming speed over the seven days of training were analyzed using a mixed ANOVA with day as a repeated measure and genotype and treatment as between-group factors. For the probe trial, the time spent in a virtual disc (27 cm diameter) centered in the target quadrant (target) and the average time spent in equivalent virtual discs centered in each of the other three quadrants (non-target) were analyzed using a mixed ANOVA with treatment group and genotype as between-group factors and quadrant (four quadrants identified as target, adjacent-clockwise, opposite, adjacent counterclockwise) as a within-subjects measure. Additional measures in the probe trial (path length, thigmotaxis time, floating time, and speed) were analyzed with factorial ANOVAs with genotype and treatment as between-subjects factors. As acquisition training progressed, five of the 46 mice began to adopt a floating posture upon being placed in the pool that persisted for large portions of most trials (>30 sec) of all subsequent training sessions (two trisomic mice given PBS, one trisomic given EGCG, and two euploid mice, one given PBS and one given EGCG). These five were excluded from the MWM analyses. On the probe trial, one trisomic mouse (given EGCG) that had completed acquisition training in typical fashion, subsequently adopted the floating posture for 42 seconds of the 60-second probe trial and was excluded from the probe analysis.

#### Brain and bone measures

The normalized DYRK1A values (as a proportion of the euploid-PBS control mean) were analyzed by two-way ANOVAs (separate for each brain region) with genotype and treatment as between-subjects factors. For skeletal measurements, the measures that were not normally distributed were transformed to achieve normality. The bone parameters (Tables 6 and 7) were analyzed with two-way ANOVAs using genotype and treatment as between-subjects factors.
